# Online reported women’s experiences of symptomatic pelvic organ prolapse after vaginal birth

**DOI:** 10.1186/s12905-019-0830-2

**Published:** 2019-10-29

**Authors:** Maria Mirskaya, Eva-Carin Lindgren, Ing-Marie Carlsson

**Affiliations:** Department of Health and Welfare, Halmstad University, SE-823, SE-301 18 Halmstad, Sweden

**Keywords:** Fertile women, Pelvic organ prolapse, Pelvic organ prolapse symptoms, Psychological trauma, Qualitative research, Vaginal birth

## Abstract

**Background:**

Pelvic organ prolapse (POP) is a major public health problem with a relative high lifetime risk of surgery. The main risk factor for developing POP is vaginal birth. Many women become symptomatic later in life and most of the existing research on symptomatic pelvic organ prolapse (sPOP) predominantly concentrates on the effects of the condition on postmenopausal women. However bothersome symptoms of POP can be reported as early as in women’s 20s and may occur shortly after vaginal birth. Limited studies provide an insight into daily life of fertile women with sPOP. Thus, we aimed to explore fertile women’s experiences of symptomatic pelvic organ (sPOP) after vaginal birth.

**Methods:**

An inductive, text-driven approach was taken by selecting thread posts from an internet forum written by 33 Swedish fertile women who had experienced sPOP after vaginal birth.

**Results:**

The overarching theme “being irreparably damaged” was identified as representing an experience of being disabled by sPOP after vaginal birth. The fertile women experienced that their lives were ruined because of physical and psychological limitations caused by this unexpected, unfamiliar and unexplained condition. Living with sPOP impinged on sexual health, restricted daily and sports activities and affected the women’s ability to fulfill everyday parental duties. This in turn compromised women’s psychological health. In addition, the negligence of healthcare professionals who tended to trivialize and normalize the symptoms led to the belief that there were no sustainable treatments and that women would have to live with bothersome symptoms of POP for rest of their lives.

**Conclusions:**

This study found that sPOP had a significant negative impact on fertile women’s lives. The women indicated that they had not had the opportunity to voice their concerns and had not been taken seriously by healthcare professionals. It is of the utmost importance to acknowledge this problem and develop guidelines for prevention and management of sPOP to improve the quality of life for women.

## Background

Pelvic organ prolapse (POP) is characterized by a weakness of the vaginal walls, resulting in descent of pelvic organs through the vagina [1]. POP has become a major public health problem with 12.6% lifetime risk for surgery in U. S [2] and 19% in Australia [3]. Some women have no symptoms despite the anatomical changes while others experience symptoms such as pelvic pressure and difficulties with urination and defecation [1]. The main risk factor for developing POP is vaginal birth which more than doubles the risk for symptomatic POP (sPOP) compared to women undergoing Caesarean sections [4, 5].

Moreover, there is an established relationship between a more advanced stage POP and postpartum levator ani muscle (LAM) avulsion [6, 7]. It is well documented that there is a decades-long delay between childbirth and onset of sPOP, and since women become symptomatic later in life [3, 4, 8, 9] it is not surprising that most of the existing studies on POP focus on postmenopausal women or women shortly before menopause [10]. Bothersome symptoms of POP can be reported as early as in women’s 20s, may occur immediately after vaginal birth [11] and may be accompanied by LAM injury [12].

Thus, the aim of this study was to explore fertile women’s experiences of symptomatic pelvic organ prolapse after vaginal birth.

## Methods

### Design

For this study, a retrospective text analysis with an interpretive qualitative approach was chosen [13]. Texts from posts within a thread from an online forum were collected since this can be used as a rich source of empirical data [14, 15].

The current study was approved by the Regional Ethical Review Board Sweden (Dnr 2017/65). All materials published on the forum are public and no password is required to read thread posts. However, registration is required for writing own posts and communicating with other members. Written informed consent to undertake the study was obtained from the forum’s web **operations manager**. An information letter with details about the study’s aim and structure and about confidentiality, the voluntary nature of and right to withdraw at any time without explanation was sent to the forum manager to be published on the forum’s website.

### Context

This study was performed in Sweden where primiparous and their partners are offered antenatal courses that includes both childbirth and parental information. A majority of Swedish women give birth at hospitals and are offered a postpartum check-up, 6 to 12 weeks after birth. These checkups should include a vaginal examination, pelvic floor assessment and exercise advice.

### Data collection

Our sampling strategy was purposive and obtained women’s posts from a Swedish online forum focusing on family life and parenting.

The forum thread was identified through the search engine Google. The specific targets were threads consisting of chains of questions and answers or simply comments posted by women who had experienced sPOP after vaginal birth. Data was collected between January 2015 and July 2017. To be included in the study, the thread posts needed to meet the following inclusion criteria: to be written in Swedish by fertile women 18 years and older who had experienced sPOP after vaginal birth. Posts which contained discussions about the postoperative recovery process after POP surgery were excluded. In total, 487 thread posts written by 54 women were read in order to obtain sufficient data. Finally, posts written by 33 women were considered relevant and selected for analysis. Information on participants was extracted from the posts. Age could be identified for 17 women, with ages ranging from 24 to 45 years old (mean = 27). 18 of the participants were primiparous and their children were mostly younger than 8 years of age. 12 of the women had children younger than 1 year old (Table 1).

**Table 1:** Background characteristics of the participating women when the data collection started

	*N* = 33		
Age (mean)	27		
Min-max	25–45		
Unknown	16		
No of Children			
One	18		
Two	9		
Three	2		
Four	2		
At least one	2		
Partner		Separated after POP	
In relationship	14		
Single	3	1	
Unknown	16		

### Data analysis

In line with qualitative content analysis, which implied moving from the data to a theoretical understanding, the data-analysis procedure followed five steps [16–18]. First, all texts from the thread posts were read several times as open-mindedly as possible to obtain an overall impression of women’s experiences of sPOP. Following this, the texts that met the study’s aim were copied to a separate document. Secondly, the texts were divided into units of meaning (segments of text or simple quotes). Thirdly, these units of meaning were condensed into keywords or key phrases that reflected the content, and coded. Fourthly, similarities and differences between codes were compared and sorted into tentative categories. The tentative categories were then reviewed and discussed and the final system of four categories with 10 subcategories was constructed. The data analysis procedure moved from descriptions of the content on a manifest level in sub-categories, to the more abstract level where the categories were established [16], and finally, in the fifth step, the four categories were abstracted into one theme (Table 3). While this analytic procedure may look like a linear process, it actually involves going back and forth between decontextualization and contextualization, which, although complex, creates analytic depth.

The first author (M.M.) in collaboration with the third author (I-M.C.) mainly performed the analysis. However, all steps in the analytical process were discussed among all co-authors until agreement was reached. An example of the process of creating of units of meaning, codes, sub-categories and categories is given in Table 2.

**Table 2:** An example of the process for creation of meaning units, codes, sub-categories and theme

Meaning unit	Code/condensed meaning unit	Sub-category	Category	Theme
*“I had quite a big cystocele, it almost bulged outside … I had difficulty emptying my bladder, always needed to pee, and I got a urinary tract and kidney infection as a result. I had to insert my fingers into the vagina and push against it when doing a poo to help to evacuate my bowels.” (Participant 3).*	*Disturbance of urinary function and disturbed defecation restrict everyday life./* *Difficulties in emptying bladder, always needed to pee got a urinary tract and kidney infection. I had to help to evacuate my bowels.*	*Being dysfunctional*	*Restricted life*	Being irreparably damaged
*“I don’t get any clear information from the doctors... They think I am young and they don’t want to cause bigger problems.......What do they mean by bigger problems??? Is an operation to the back wall so complicated?? Do you become so tight? And do most of them become so much worse after surgery at the front that they pee themselves?”* (Participant 4).	*Lack of proper information* *contributes to* *experience of being neglected by healthcare* *I don’t get any clear information from the doctors...*	*Lack of information*	*Neglected by healthcare*	

## Results

### Being irreparably damaged

The overarching theme “being irreparably damaged” illustrates that the women found themselves in a hopeless situation balancing being a parent and being disabled because of sPOP. Ambivalent feelings of happiness about being a mother were often expressed and at the same time the women experienced feelings of hopelessness at being far too young to be living with a nonfunctional body.

When accessing healthcare, some of them were told that surgery was not an option since they were too young to undergo a procedure that could cause even bigger problems in comparison with their current situation. This development, meaning that they would have to live with sPOP for the rest of their lives, left them in an impossible situation. Phrases like “ruined life” and “wanting my life back” revealed the women’s sense of being damaged for life.

*“But perhaps it is the case that people can’t even understand how terrible it feels to have wide-open, aching vagina. It totally crushes your life. You can’t walk, stand or sit properly. Can’t have intimacy with your partner or even pee or poo normally. It is awful.”* (Participant 3)

The theme “being irreparably damaged” consists of four categories: taken by surprise, restricted life, neglected by healthcare, and psychological intrusion, with additional subcategories (Table 3).

**Table 3:** The overall theme “being irreparably damaged” with four categories and additional sub-categories

Sub-categories	Categories	Theme
*Not knowing their body*	Taken by surprise	Being irreparably damaged
*Not being warned*		
*Being dysfunctional*	Restricted life	
*Having constant discomfort*		
*Being non-sexual*		
*Worry about worsening*		
*Lack of information*	Neglected by healthcare	
*Being trivialized*		
*Distorted body-image*	Psychological intrusion	
*Being mentally broken*		

### Taken by surprise

There was a general unawareness of the existence of the condition ‘pelvic organ prolapse’ until the first symptoms were discovered. The feeling of being taken by a dreadful surprise was prominent in the women’s stories. This consisted of surprise at not knowing their own bodies and a lack of warning that something like this could happen.

#### Not knowing their body

Unfamiliarity with pelvic floor anatomy was frequently described, regarding both the normal anatomical position of the uterus and the appearance and structure of the vaginal walls. Not knowing what constituted normal anatomy made it difficult for the women to understand the abnormalities they now had to contend with.

*“…I have personally asked about my cervix, which I think is sitting low since giving birth … I wonder how you know whether it is a lateral defect and what it means? And I personally have no idea of how the vaginal walls should look/feel when they are “normal” and I don’t know where a cervix should be positioned, I just haven’t thought about that kind of thing before.”* (Participant 2)

#### Not being warned

Vaginal birth was the norm of discourse and considered the normal way to give birth, and no one had told the women that it might be associated with an increased likelihood of injury. The women expressed that they had not been warned during prenatal education courses, which caused feelings of being misled. Anger and astonishment arose, vaginal birth was described as a fatal mistake, with many women saying that if they were to give birth again they would prefer to undergo a Caesarean section. One woman went as far as saying she would not have had children if she had known about the risks.

*“Everything is just so normal, if I had known before what would have been normal, I would probably not have had children or fought for a Caesarian! Nope, it is impossible to imagine the consequences these problems lead to. Would be easier to have an injury in absolutely any other body part!”* (Participant 7)

### Restricted life

The women experienced that sPOP restricted their lives dramatically, which they had to adapt to. Their daily lives were seriously affected by a dysfunctional body, constant discomfort, and worrying about sPOP worsening from both a short- and a long-term perspective. Moreover, sPOP also impinged on intimate relationships.

#### Being dysfunctional

Disturbance of urinary function and defecation had a negative impact on everyday life. The women described several difficulties such as altered micturition and defecatory patterns, including symptoms of urinary urgency, frequent urination, urine retention, voiding problems, urinary incontinence, urinary tract infections, and difficulties emptying the bowels. What previously had been an ordinary physical function did not work anymore, which impinged on every aspect of life, including employability. The women had become desperate in their attempts to overcome these problems, for instance by fasting in order to avoid emptying the bowels or overdosing on laxatives to avoid being constipated. Furthermore, they used various techniques in order to facilitate bowel emptying, for instance the need to splint.

*“I had quite a big cystocele, it almost bulged outside. I had difficulty emptying my bladder, always needed to pee and I got a urinary tract and kidney infection as a result. I had to insert my fingers into the vagina and push against it when doing a poo to help to evacuate my bowels.”* (Participant 3)

#### Having constant discomfort

The women described feelings of constant discomfort which severely disrupted daily life, such as pressure, heaviness, fullness, and the sensation that something was falling out of the vagina. Some women complained about vaginal flatus, and an awful “open” feeling with a dragging sensation in the vagina. Some women also spoke of itching, soreness in the vagina and rectum, and lower back and abdominal pain.

*“ … I am probably also most upset about being so open. Feels like I have something in the way when I go to the toilet, I want to feel to see if something has fallen down but, each time I feel, everything seems to be in the right position. I also take in a lot of air. I go around queefing to get the air out, I feel really stupid….”* (Participant 17)

This constant discomfort was compounded by basic day-to day activities such as cooking and walking, and even standing or sitting. The women described an inability to use tampons because of a prolapsed uterus, which prevented them from doing exercise and other physical activities.

#### Being non-sexual

Living with sPOP caused various sexual health problems such as painful intercourse, lack of sexual pleasure and inability to achieve orgasm. Sexual intercourse no longer provided any pleasure for the women and they complained of decreased vaginal sensation and vaginal looseness. Women described that they had to adjust to specific positions during intercourse or had to discontinue because of pain and unpleasant feelings. These limitations made sex less enjoyable and resulted in faked orgasms, having sex only for their partner’s sake, and long intervals between intercourse or inability to have penetrative sex. A nearly non-existent sex life resulted in feelings of being non-sexual, disabled and destroyed as a woman. The women described that they wanted badly to be more sexually active like they used to be before they had had a child, which caused sadness and grief over the loss of sexuality.

*“I also have problems with my pelvis area 2 years after having my baby. … it hurts during intercourse so we more or less don’t have a sex life… I stopped feeling like it because of all my genital troubles…. I mourn that…”* (Participant 11)

Moreover, the women commonly expressed fear of abandonment by their partner due to the inability to have sex. Ideas about allowing their partners to have sex with someone else just for the relationship’s sake were discussed in the thread.

*“At some point he will feel sad about not being allowed to have sex. Should I then let him go out with other people or just be alone?”* (Participant 27)

#### Worry about worsening

The women were worried about sPOP worsening. This restricted them as they lived in permanent fear that doing regular household tasks like lifting groceries, or activities that were considered simple parental responsibilities like playing with children, might worsen the symptoms. Having to renounce activities and constantly worrying about making things worse led to frustration, anger, sadness and a feeling of being inadequate mothers.

*“… I don’t dare lift heavy things, become constipated or exercise etc. I am also young (26) …I can’t help my 3-year old up onto the swing … it hurts my heart so much: ( . My little girl, who is a month old, already feels heavy to carry … how will I be able to help my children and take care of them? How will I manage my everyday life with two small children? I want to play with my children and feel a sense of freedom in my body.”* (Participant 23)

In a long-term perspective, the women feared the future and especially the potentially worsening symptoms of their condition due to the menopause and the ageing process. Moreover, the women expressed that they were afraid of being further damaged following another pregnancy and birth, or, if they had undergone surgery, compromising this if they were to give birth again.

*“Will I be able to have children without destroying my body even more?… I’m so worried and now my partner wants children and I am so afraid! Afraid about destroying my life.”* (Participant 9)

### Neglected by healthcare

There was a discrepancy between women’s experiences of their problem and how they were treated by the healthcare system. This discrepancy was considered as due to the fact that healthcare professionals demonstrated inability to give proper information, but also a tendency to trivialize and neglect both the condition and the women. This negligence reduced opportunities to get help and led to feelings of not being taken seriously.

#### Lack of information

It was considered important to receive a proper diagnosis, treatment and psychological support, which was sought from different types of healthcare providers. However, the women frequently did not receive proper information. For example, some received completely different diagnoses from different physicians and physiotherapists. Postnatal healthcare was described as a catastrophe, and women expressed shock at how little some healthcare professionals knew about sPOP. Some women stated that they had been convinced to give birth vaginally and now that they had been damaged, no-one took responsibility or had the competence to deal with the problem. The women wanted to know more about the condition to prevent the damage getting worse, such as advice on lifestyle changes and how to adjust physical activity in relation to sPOP. However, it was found difficult to access good, unambiguous information. Instead, information from doctors was inconsistent, unclear and difficult to understand, and advice was not in line with what women found out by themselves from independent sources. This left them with more questions than answers.

*“I don’t get any clear information from the doctors… They think I am young and they don’t want to cause bigger problems … What do they mean by bigger problems? Is an operation to the back wall so complicated? Do you become so tight? And do most of them become so much worse after surgery at the front that they pee themselves?”* (Participant 4)

#### Being trivialized

Women felt that sPOP was a condition that was trivialized and that the healthcare providers acted as if the women were “making a big thing out of it”. There were often discrepancies between the physician’s account of signs and symptoms and the women’s own experiences. Physicians explained sPOP as a natural condition after vaginal birth, using expressions that belittled and normalized the problem like “it doesn’t look too bad”, “minimal collapse of front wall”, “nothing abnormal” and that “POP is very common”. During the pelvic examination, the physician compared the woman with other patients and comment that “he had seen worse”. However, contrary to healthcare professionals’ opinions, women did not perceive the symptoms of POP as normal or natural, and this attitude was experienced as humiliating and discouraging.

### Psychological intrusion

Women expressed that sPOP caused psychological harm and distress in multiple ways such as distorted body-image and negative feelings toward their own genitalia. Further, living with sPOP invaded them mentally with intense grief and anxiety.

#### Distorted body image

sPOP negatively affected the women’s body image and they saw no beauty in themselves. Living with sPOP was seen as having “an old lady’s disease”, and feelings of disgust and embarrassment with themselves were highlighted in the thread. Comparing themselves before and after birth nurtured a negative attitude and an overt dissatisfaction towards their own vagina and difficulties accepting the new appearance of their genitalia, as one woman expressed:

*“I feel disgusting and have difficulty accepting how I look.”* (Participant 6)

#### Being mentally broken

Women expressed that sPOP had broken them mentally, and the psychological impact of sPOP was often considered even worse than the physical problems. All their thoughts were focused on their bothersome symptoms. Anxiety and panic attacks were common and could be induced by simple everyday things like toilet visits. The happiness of becoming a parent was overshadowed by the feeling that they had become disabled because of sPOP. Moreover, an inability to participate in activities they had enjoyed before, such as high-intensity sports or a job as a training instructor, which were a key part of the women’s identity, had the potential to cause an identity crisis, leading to depression.

The psychological impact of sPOP caused women to take sick leave due to depression and some were put on antidepressants. Forum members expressed the feeling that sPOP had turned their life into a living hell, and some of the women even experienced suicidal thoughts.

*“I cry during all my spare time when my partner isn’t home… I am so depressed that I can hardly cope with life…. I would rather not live any longer but need to for my son… Thoughts of suicide… Feeling of not wanting to live any more…. I ask myself how to cope with this hell every bloody damned day…Want to be happy… Soon won’t be able to take any more…Living with thoughts about my vagina 99% of the day… (Participant 11).*

Despite the psychological distress, women had to cope with the condition and the forum helped them to keep looking ahead in life. Sharing helpful tips and happy personal stories about good outcomes of surgery, and just being a part of a group that reminded them that they were not alone, encouraged the women and made them feel better.

## Discussion

This study demonstrates that sPOP has profound consequences for the fertile woman and her family. The women in our study revealed that they had been irreparably damaged and felt that their lives had been ruined. We argue that our study brings women’s subjective experiences into focus and highlights the importance of considering experiential knowledge without exception when evaluating and treating sPOP. The women perceived negatively the interactions with healthcare professionals while encountering care. Normalization, trivialization and belittling of the condition were common where healthcare professional authoritative knowledge was valued over the woman’s experience.

In addition, the women felt dissapointment not only with postnatal care but with prenatal education. They wondered why no one informed them on potential risks prior the delivery. Improved information about potential pelvic floor problems could help better prepare women to manage sPOP following vaginal birth. Sadly, several other studies have shown similar results as ours regarding miscommunication between women with pelvic floor injury and healthcare professionals [11, 12, 19, 20]. Midwives, obstetricians and physiotherapists need to be aware of the discourse on these forums and take part in the dialogue in order to ensure professional support for these women.

The women in our study suffered from multiple symptoms of urinary and bowel dysfunction and felt constant discomfort and pain, in line with experiences described in previous research [11, 12]. This in turn impinged on their sexual health and sexuality such as orgasm, intimacy, relationship satisfaction and physical ability to engage in penetrative sex. The women emphasised a difficulty to achieve orgasm and lack of vaginal sensation due to vaginal looseness, which is in contrast with previous research claiming that sPOP has no effect on orgasm and sexual satisfaction [21]. Our result may be due to the data being from an online forum where the women could remain quite anonymous [15]. Emotional responses to altered sexual function, as reflected in feelings of being a disabled and destroyed woman, are not surprising and are in concordance with other studies [11, 12, 22]. In summary, the women experienced that sPOP had turned their lives into literally a living hell; they reported suicidal thoughts and feeling of hopelessness and tended to have a bleak outlook on the future. Thus, we can observe the link between onset of sPOP after vaginal birth and psychological trauma, which is in line with an Australian study [12].

Although there is a growing awareness of earlier unrecognized childbirth-related pelvic floor injury like LAM avulsion [6], the bothersome symptoms of POP are still viewed by healthcare providers as normal expected unavoidable issues after vaginal birth and are not investigated properly. Healthcare professionals seem to be unaware of the real impact of this condition on the quality of life and psychological health of new mothers. The Swedish Agency for Health Technology Assessment and Assessment of Social Services reveals that a large number of treatments given to women with childbirth traumas have not been scientifically evaluated in reliable systematic reviews, and there are no systematic reviews regarding addressing the acute POP associated with vaginal birth [23]. It may be assumed that this lack of evidence, can partly explain our findings that the healthcare professionals’ failed to meet the women’s needs regarding diagnostic, information and treatment options.

The strength of this study is that qualitative method was used to provide insight into fertile women’s daily lives from the perspective of those who experience the problem. Our findings highlight the condition as a *worthy topic* [24] complementing the existing quantitative research proposed that maternal birth trauma is a public health problem [25]. *Rich rigour* [24] was enabled by use of data that covered a time period of 2 years and 7 months with 33 participants, which generated a wide variation of experiences. Online posts, compared to interviews, can sometimes be more detailed than verbal narratives, particularly in discussions about body parts and sexual issues [15]. On the other hand, a limitation was that some of the posts were fragmentary and it was not possible to obtain additional information by asking questions, for instance, about the grade of POP or proposed treatment plan, which is a known disadvantage of online data collection [15]. Furthermore, bloggers may comprise an atypical sample of the population. It could be assumed that women who seek support on the internet forum might have bad experiences encountering healthcare and this may have caused a selection bias by an opinionated group who already have a position on the issue*.* Women who had positive experiences could have been overlooked. Therefore, our finding may not reflect the experiences of women with good pelvic floor recovery postpartum and with good outcomes of conservative treatment. The other limitation applies to sexual difficulties experienced by women. It should be taken into consideration that the demands of childcare and psychological trauma accompanying childbirth could also contribute to lack of sexual desire and performance.

Additionally, the current study has shown similar results as other qualitative studies from Netherland, Australia and USA [11, 12, 22] which could demonstrate transferability of the results [24]. As an aid to increase the *credibility* of the study, a variety of voices from the participants are included with rich quotes in the findings. Trustworthiness is strengthened by the fact that a group of interdisciplinary researchers with a lot of experience from qualitative research performed the analysis via discussions until consensus was achieved [17].

## Conclusion and clinical implications

This study demonstrates that sPOP has a significant impact on fertile women’s lives. The women expressed that they had been irreparably damaged which restricted and ruined their lives, as well as impacted negatively on the whole family. The women indicated that they had not had the opportunity to voice their concerns and had not been taken seriously by healthcare professionals. It is of utmost importance to address these issues after delivery in follow-up counselling to acknowledge the problem. Symptoms of POP after vaginal birth should be included in existing Pregnancy and Medical Birth Registers as well as prenatal consent forms that highlights the risks of vaginal birth and traumatic LAM injury. Furthermore, there is a need to develop national guidelines for prevention and management of sPOP.

## Data Availability

The data that support the findings of this study are available from Halmstad University but restrictions apply to the availability of these data, which were used for the current study, and so are not publicly available. Data are however available from the authors upon reasonable request and with permission of Halmstad University.

## Abbreviations

LAM: Levator ani muscle
POP: Pelvic organ prolapse
sPOP: Symptomatic pelvic organ prolapse
